# Cathodoluminescence Enhancement Mechanisms in Silica Microspheres

**DOI:** 10.1002/nap2.70055

**Published:** 2026-03-24

**Authors:** Hadar Aharon, Zahava Barkay, Sophie Meuret, Ofer Kfir

**Affiliations:** ^1^ School of Electrical and Computer Engineering, Iby and Aladar Fleischman Faculty of Engineering Tel Aviv University Tel‐Aviv Israel; ^2^ Jan Koum Center for Nanotechnology and Nanoscience Tel‐Aviv University Tel Aviv Israel; ^3^ CEMES–CNRS Toulouse France

**Keywords:** cathodoluminescence, microspheres, whispering‐gallery modes

## Abstract

Cathodoluminescence (CL) enables optical‐frequency analysis of samples with nanometer resolution, originating from the interaction of a focused electron beam with radiative electronic states or directly with the optical modes of the sample. Here, we decompose the various mechanisms underlying CL generation and emission from an archetypal spherical resonator using its spectral, angular, and spatially resolved features. We investigate the radiation of optical whispering‐gallery modes in regimes of coherent and incoherent luminescence. The use of different experimental regimes allows us to disentangle the different contributions to the CL in spheres, namely photon absorption, generation, and radiative leakage, and conclude that the photon generation occurs precisely on the sphere's surface. In addition, the spheres serve as high‐NA collimating lenses for CL, resulting in a mode quality unprecedented for CL in free space. We believe that such collimated and directed CL in free space will enhance existing quantum measurements of CL and facilitate new ones, such as high‐rate electron‐photon entangled pairs, CL from quantum emitters, and homodyne analysis of CL.

## Graphical abstract

Microspheres are prototypical optical resonators that affect cathodoluminescence (CL) in their surroundings. By resolving CL spectrally, spatially, and angularly, we disentangle the photon generation, absorption, and radiation mechanisms. We find two regimes: The first is dominated by CL from whispering gallery modes, which emanates exclusively from the surface. The second is CL collimation, forming high‐quality beams, which is particularly suitable for quantum optics measurements.

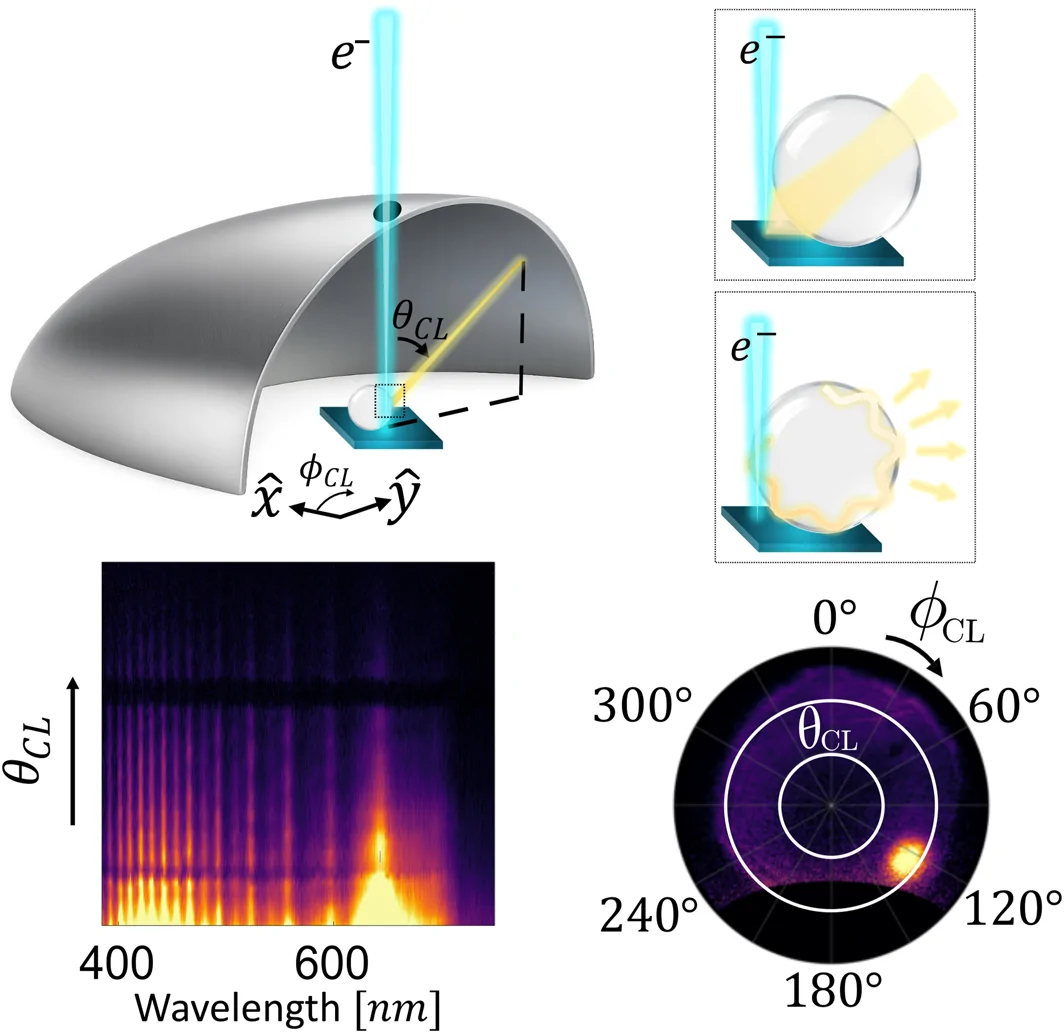

## 1 Introduction

Electron microscopes are ubiquitous analytical tools for the ultrasmall, enabling imaging, diffraction, and spectroscopy of materials down to the atomic scale [1, 2, 3]. The ability to pinpoint the electron beam with an accuracy well below an optical wavelength is used to quantify local optical responses that would otherwise be smeared by the light diffraction limit [4, 5, 6, 7], such as the mapping of evanescent fields, dark modes [8, 9], high momentum states [10, 11, 12], and more. Capturing photons emitted from an impinging electron, namely cathodoluminescence (CL), was applied to characterize plasmonic nanocavities and surface plasmon‐polaritons [13, 14, 15, 16, 17, 18, 19], resonant dielectric nanostructures [20, 21, 22], mineral coloration in geoscience [23, 24], thin films in semiconductors [25], etc., providing access to their spectral, spatial, polarization, and angular‐resolved emission properties [26, 27, 28]. An alternative approach for probing photon generation instead of emission is EELS [29, 30], which stands for electron energy loss spectroscopy, used in a complementary manner to probe the correlations and entanglements formed by the electron‐photon coupling [31, 32, 33, 34, 35, 36]. The high resolution of CL is particularly powerful for analyzing the properties of discrete modes within optical resonators. By exciting the sample with a focused electron beam, CL probes the local density of optical states (LDOS) of photonic crystals and defects, both spatially and angularly [22, 29, 37, 38, 39]. For example, CL spectroscopy has been used to study one‐dimensional pillar resonators [40], angle‐resolved CL in plexcitonic crystals has revealed coupling‐induced modifications of the band structure and a redistribution of optical states between loss channels [41, 42], and in topological photonic lattices, CL has directly probed the LDOS of bulk and edge states while visualizing their spatial field distributions [43]. Similar approaches have also been applied to optical whispering‐gallery modes (WGMs) circulating on the edge of dielectric microcavities [21, 32, 44, 45, 46, 47]. These modes are typically characterized by global properties such as their quality factor (Q‐factor, or Q), modal volume (V), polarization (TM or TE, for transverse magnetic or electric polarization), radiation loss, and absorption [44, 48]. But in the context of their interaction with an electron and the generation of CL, local properties become important, as well as the particular coupling mechanism. One can divide the electron‐sample interaction into coherent and incoherent processes. Coherent CL stems from integrating over the electron trajectory in space and time in a manner that matches the dynamics of the optical mode's field, sometimes referred to as phase matching. It includes transition radiation [26, 27, 49, 50], Cherenkov radiation [39, 51, 52, 53, 54], and Smith–Purcell radiation [55, 56]. In incoherent CL, the generated charge carriers populate intrinsic material excitations, such as defects, excitons, and higher bands [57, 58, 59], etc., followed by spontaneous emission [22], for which the phase is undetermined. In practice, the generated CL photons need to reach a detector, which typically requires their out‐coupling in its direction, making the analysis of optical cavities more complex. On one hand, a good resonator may have a high Q‐factor and density of states, but the confinement of light to it inherently means that radiation to the environment is inhibited. On the other hand, a poor resonator would emit broadly spectrally and angularly, suffering from a low signal‐to‐noise ratio, and may be dominated by other optical signals of the sample. These are particularly harsh tradeoffs for quantum optical measures, where one ideally wants a narrowband emission with a well‐defined radiative mode [60, 61, 62, 63, 64].

In this work we investigate the CL properties and enhancement mechanisms of individual silica microspheres. We use a focused electron beam in a scanning and in a transmission electron microscope (SEM and TEM) with beam energies of 30 and 200 keV, respectively, and use spheres with several diameters. We show that different photon generation and radiation phenomena are separable spatially and angularly. Varying the electron beam energy and the sphere's diameter disentangles CL generation mechanisms, radiative efficiency, and material absorption. As an example of competing luminescence phenomena, we address the collimation of external CL, showing that its dominance under some parameters may hinder the analysis or be used as a beneficial feature for out‐coupling CL with a good spatial mode.

## 2 Results

As our main experimental platform, we use an Apreo 2S‐LV SEM by Thermo Fisher Inc., retrofitted with the SPARC CL analyzer and a parabolic collection mirror with a numerical aperture (NA) of 0.98 from Delmic Inc. (see Figure 1). The parabolic mirror projects the angular distribution of the CL directly onto the camera. Alternatively, a θ‐resolved spectrum is captured by placing a vertical slit parallel to the azimuthal angle ϕ=0°. The polar angle θ is mapped onto the vertical axis of the camera (negative values for ϕ=0° and positive values for ϕ=180°), and the wavelength spans the horizontal axis.

**FIGURE 1:**
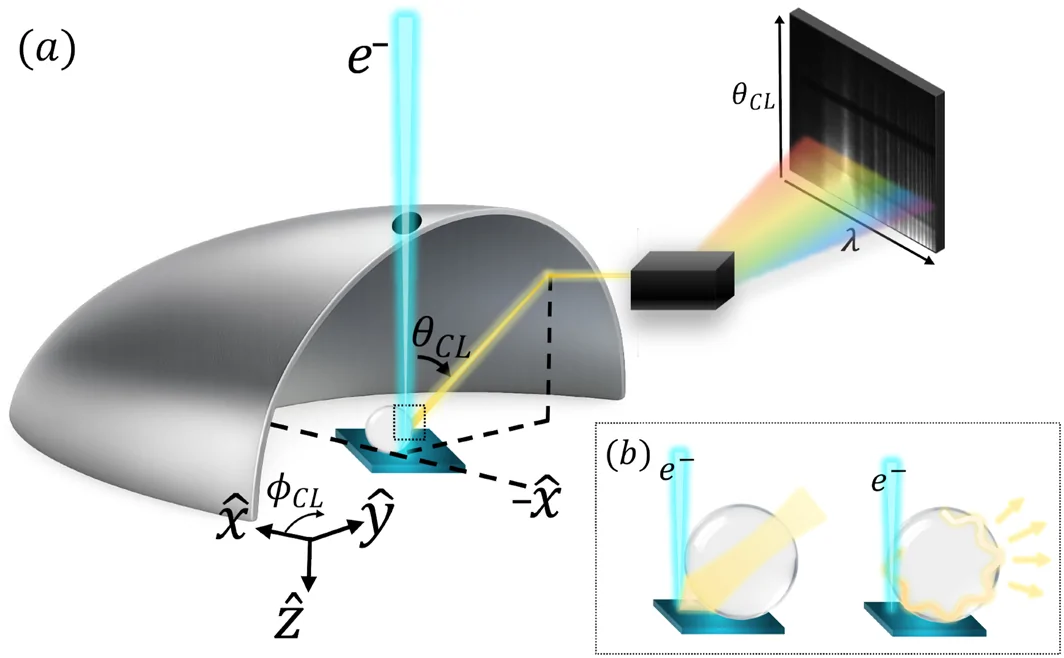
Schematic of the measurement setup. (a) An electron beam is focused on the sample, generating CL photons, which are collimated through a parabolic mirror and mapped to a spectrum versus the polar angle $\theta_{CL}$, around $\phi_{CL}=0{}^{\circ}/180{}^{\circ}$. (b) The inset shows CL enhancement by the excitation of a resonant WGM (right) and by geometrical beam collimation (left).

We analyze CL generation and emission mechanisms in silica glass microspheres with diameters of 2.1 μm, 4.4 μm, and 62 μm, as shown in the secondary‐electron micrographs in Figure 2a–c, acquired in the SEM at an acceleration voltage of 30 keV. The electron‐beam position is marked by an orange circle in Figure 2a–c. The 62 μm sphere, fabricated from a tapered optical fiber, exhibited no clear resonances in the CL, and spectra from the 4.4 μm sphere are discussed later. The smaller two are silica microspheres (Bangs Labs, SSD5003 and SSD4002), provided as a dry powder. The powder was dispersed in isopropanol and drop‐cast onto a standard 3‐mm TEM grid coated with a 20‐nm thick lacey carbon film (TED Pella 01841 and 01894). Figure 2d presents a θ‐resolved spectrum acquired with an integration time of 30 s from the 2.1 μm sphere. The corresponding raw data are shown in the Supporting Information S1: Figure S4c, with additional angular‐resolved examples. The purple spectrum in Figure 2e is integrated over the angular range $-70{}^{\circ}\leq \theta_{CL}\leq -10{}^{\circ}$, which is the widest range within the low background region of the spectrogram. The orange spectrum is from the range $-79.4{}^{\circ}\leq \;\theta_{CL}\leq -77.6{}^{\circ}$, in which competing luminescence phenomena decrease the peak contrast. Across the acquisition band, we identify 17 distinct spectral peaks corresponding to WGMs, nearly independent of the emission angle. Indeed, the emission of incoherent WGM is expected to be omnidirectional [65] (see Supporting Information S1: Figure S4 for various beam positioning).

**FIGURE 2:**
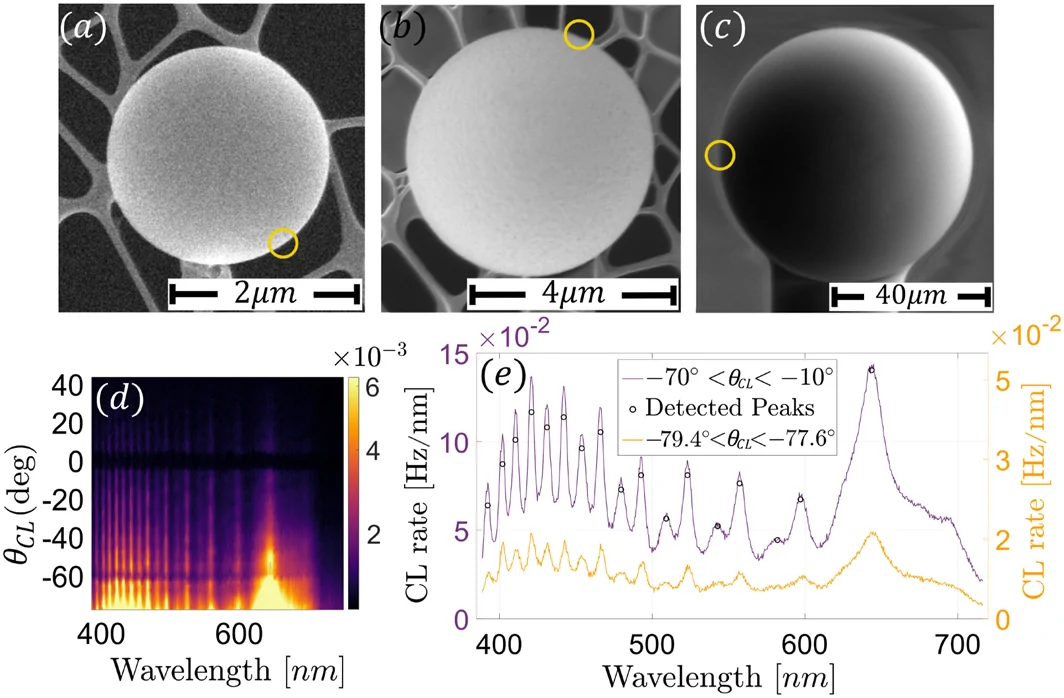
Angular‐resolved CL spectrum. (a)–(b) SEM images of silica microspheres with diameters of 2.1 μm, 4.4 μm, respectively. The electron‐beam position is marked by an orange circle. (c) SEM image of a 62 μm diameter sphere fabricated from a tapered optical fiber. (d) θ‐resolved CL spectrogram from the 2.1 μm sphere, showing peaks of the sphere's WGMs with a spatially varying background. (e) CL spectral count rate extracted from (d), summed over a low‐background range $-70{}^{\circ}\leq \theta_{CL}\leq -10{}^{\circ}$ (purple) and a high‐background range $-79.4{}^{\circ}\leq \theta_{CL}\leq -77.6{}^{\circ}$ (orange).

Figure 3 shows the role of the electron‐beam position on the CL distribution from the 2.1 μm microsphere, integrated over the entire spectrum. The spatial properties of the CL are analyzed in four‐dimensions, two for the electron incidence and two for the emitted CL. The electron beam is scanned across different positions on the sphere, each described in polar coordinates within the plane perpendicular to the beam propagation, defined by a radial distance from the sphere center $r_{e}$ (the impact parameter) and an azimuthal angle $\psi_{e}$ (see Figure 3c), such that $\vec{r}=r_{e}\left(\cos\left(\psi_{e}\right)\hat{x}+\sin\left(\psi_{e}\right)\hat{y}\right)$. The angular distribution of the CL is mapped by the polar and azimuthal angles, $\theta_{CL},\phi_{CL}$ (see Figure 1). Figure 3a presents $\phi_{CL}$ versus the impact angle, $\psi_{e}$. We consider $\psi_{e}$ as an accurate parameter, whereas for $\phi_{CL}$, we present its mean. Since the physical system is rotationally invariant, the emission angle depends linearly on the impact angle, $\phi_{CL}\propto \psi_{e}$. Here, the dominant feature is that they are diametrically opposed, $\psi_{e}-\phi_{CL}=180{}^{\circ}$, due to the positive curvature of the sphere. A concave surface would overlap the impact and emission angles. For small impact radius $r_{e}$ (dark data points), the emission angles are poorly defined. It is visually evident in the spread of darker versus lighter data points, as well as in the standard deviation of $\phi_{CL}$, presented as error bars for a segment of the data in Figure 3b. Figure 3d,e show the angular CL maps for two exemplary impact positions. The rounded dark segment originates from the flat edge facet of the collection mirror. The irregular dark periphery (θ∼90°) of the angular CL is due to the roughness of the supporting copper grid, blocking CL emitted at shallow angles. Figure 3d is the emission from an electron impact right outside the sphere's perimeter $\left(r_{e}\approx R_{\mathrm{sphere}}\right)$ and resides on the linear trend $\psi_{e}-\phi_{CL}=180{}^{\circ}$ in Figure 3a. These parameters form CL that is nearly collimated, narrowly distributed in both $\phi_{CL}$ and $\theta_{CL}$. In contrast, Figure 3e shows the CL angular map of a small impact parameter ($r_{e}=0.2R_{\mathrm{sphere}}$), yielding broad and poorly defined emission angles. The corresponding data points in Figure 3a deviate from the diametrically opposed trend, albeit with a large experimental error due to their angular spread. Figures S2 and S3 in the Supporting Information S1 present explicit sets of angular CL maps alongside the corresponding electron impact parameters. The angular‐independent CL in Figure 3e is dim since Figure 3d,e are normalized separately. The omnidirectional CL emission in Figure 3e originates from WGM and will be referred to as WGM‐regime.

**FIGURE 3:**
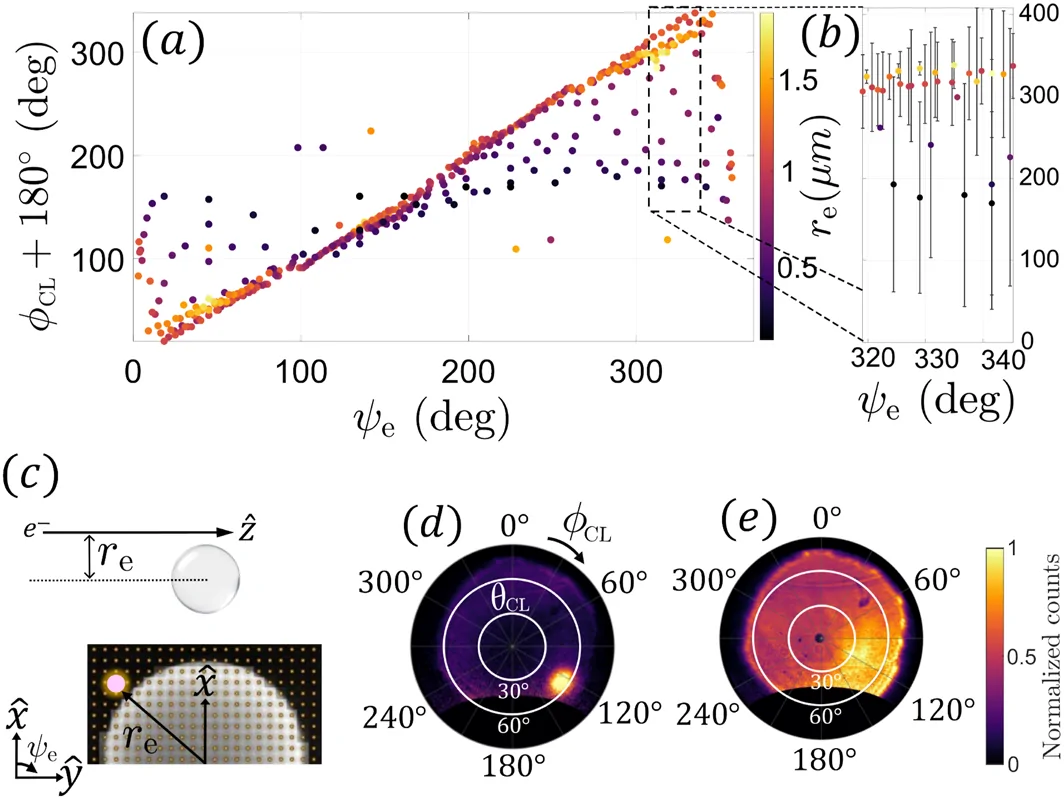
Angular dependence of CL emission on the electron impact parameter. (a) CL emission peak angle $\phi_{CL}$ as the function of the beam impact angle $\psi_{e}$, shows an emission trend to the opposing angle due to the rotation invariance and the sphere. (b) Expanded view of the marked segment, with the corresponding uncertainties. (c) Definition of the excitation coordinates and sampling grid, the electron impact position is parameterized by the radial distance $r_{e}$ from the sphere center and the azimuthal angle $\psi_{e}$, overlaid on a microscopic image of a sphere. (d) CL angular distribution for $\psi_{e}=320{}^{\circ},r_{e}=R_{\mathrm{sphere}}$ and (e) for $\psi_{e}=320{}^{\circ},r_{e}=0.2R_{\mathrm{sphere}}$.

Another regime is apparent in Figure 4, analyzing the CL's polar angle $\theta_{CL}$. There is a monotonic trend of $\theta_{CL}$ versus $r_{e}$ up to the sphere's edge. In principle, one expects that for $r_{e}\to 0$, $\theta_{CL}$ would nullify, but the CL distribution is too wide to show that clearly. However, for $r_{e}>R_{\mathrm{sphere}}$, the angular distribution shrinks, forming a sharp spatial mode as in Figure 3d. For $r_{e}>R_{\mathrm{sphere}}$, the trend in Figure 4 is no longer monotonic. Therefore, two regimes can be clearly distinguished: $r_{e}<R_{\mathrm{sphere}}$ and $r_{e}>R_{\mathrm{sphere}}$ as the WGM‐regime and collimation‐regime, respectively. CL in the collimation regime originates from outside the sphere. In our case, CL emanates from carbon; either from the laces holding the sphere (see Figure 2a,b) or from the film that covers the holes between them intermittently. Figure S3 in the Supporting Information S1 shows that no CL is detected for $r_{e}≳R_{\mathrm{sphere}}$ in the absence of an underlying layer. Thus, the sphere acts as a collimating optic for emitters on the surface underneath.

**FIGURE 4:**
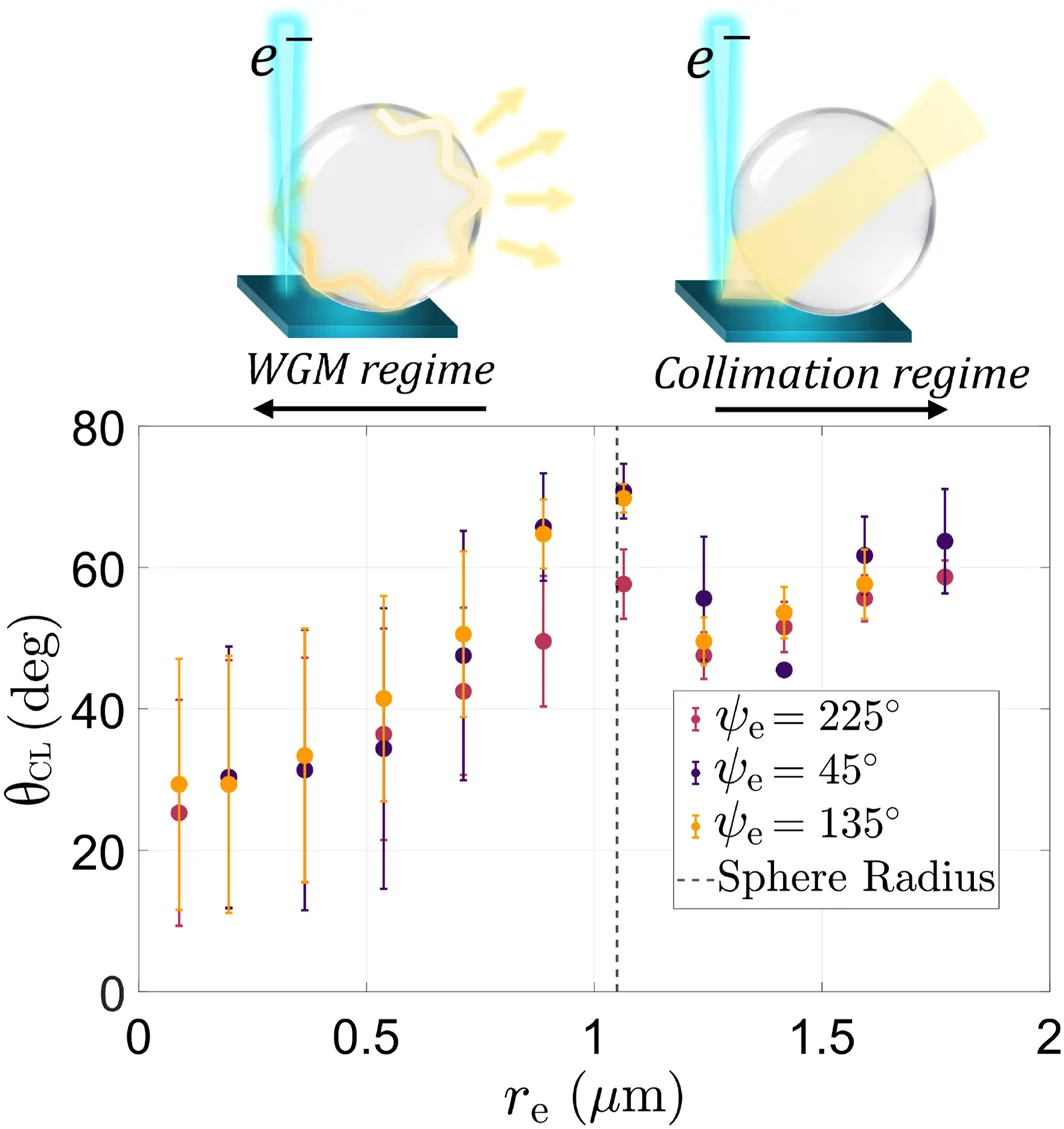
Detected mean emission angle, $\theta_{\text{CL}}$, as a function of the electron beam's impact parameter, $r_{e}$, for three azimuthal angles. The sphere's radius is indicated by the black dashed line.

In fact, the on‐sample collimation enabled by the silica sphere could be a beneficial feature for downstream applications of CL (see Figure S5 in the Supporting Information S1 for an optics ray‐tracing illustration). First, it collects a solid angle of 1.84 sr, centered at an inclination of around 45° (see illustration in Figure 1 and data in Figure 3d). For an omnidirectional emission, the collection efficiency would then be 1.84/4π=14.6%. Second, the outgoing mode has a low‐divergence, with a nearly transform‐limited Gaussian profile. The measured standard deviation of the angular distribution in Figure 3d is $\sigma_{\theta }\approx \sigma_{\phi }=5{}^{\circ}$. Assuming that the mode has an initial size equal to the sphere's diameter, we estimate that its emittance is 0.17 μm⋅rad, corresponding to $M^{2}\approx 2$ [66]. For some context, consider the constraints of the CL‐collection mirrors in TEMs, which protrude into the narrow objective‐lens gap [67, 68, 69, 70]. The collimation enables the suppression of possible aberrations in the mirror, resulting in a mode with sufficient purity for quantum measurements, or an efficient coupling to a single‐mode fiber [61, 71].

## 3 Discussion

In the following, we analyze the angular‐independent spectra to disentangle their features and evaluate the origin of the CL photon generation. The CL signal inherently convolves the contributions of the (i) generation of a photon in the resonator, (ii) radiative coupling to free space, and (iii) material absorption. To identify these components separately, we use spheres with several diameters and tune the electron‐beam energy. These degrees of freedom assist in distinguishing the different contributing phenomena and analyzing each of them in the regime where it dominates. We note that spatially filtered spectra are key, since they allow us to omit angular regions for which the CL signal is dominated by an irrelevant source of CL. In Figure 2d, the signal near $\theta_{CL}\to -90{}^{\circ}$ has a higher background due to a residue of collimated, non‐WGM luminescence.

We begin by addressing the competition between the loss mechanisms, namely photon absorption and radiation to free space, through their contributions to the quality factor of the microresonator. After addressing the loss, we analyze the generation processes of light in the resonator.

Let us consider the processes following the population of a WGM, regardless of how it was excited. The interplay between material absorption and radiation is reflected in their respective lifetimes, $\tau_{\mathrm{mat}}$ and $\tau_{\mathrm{rad}}$. These are typically expressed in terms of the quality factors, $Q_{\mathrm{mat}}=\omega \tau_{\mathrm{mat}}$ and $Q_{\mathrm{rad}}=\omega \tau_{\mathrm{rad}}$, where ω is the optical angular frequency of a chosen spectral peak. The total quality factor, which is measurable, is then

(1) $$\frac{1}{Q_{\mathrm{tot}}}=\frac{1}{Q_{\mathrm{rad}}}+\frac{1}{Q_{\mathrm{mat}}},$$

The balance between the decay mechanisms varies with the sphere's size. As the diameter increases from 2.1 μm, through 4.4 μm to 62 μm (Figure 2a–c), the radiative loss diminishes. Thus, the balance between the radiative and absorptive mechanisms in the 2.1 μm spheres shifts to an absorption‐dominated regime in the 4.4 μm spheres, whereas the CL in the largest spheres is entirely suppressed. To give a quantitative example, the theoretical radiative lifetime for TM‐polarized modes is 28 fs and $10^{4}$ fs in the 2.1 and 4.4μm microresonators, respectively. These values are calculated for similar wavelengths, 503 nm and 505 nm. The lifetime is the inverse linewidth calculated using the WGMode package for MATLAB [72], for a given refractive index (1.45) and the diameters mentioned above. For all the identifiable peaks in the spectrum, the quality factor of a peak centered at a wavelength λ is extracted from its full‐width‐at‐half‐maximum (FWHM) bandwidth, Δλ, by $Q_{\mathrm{tot}}=\Delta \lambda^{-1}\lambda$ [44]. All spectral peaks were fitted with a Lorentzian line shape using MATLAB. Representative fits are shown in Figure S1b of the Supporting Information S1, and the full fitting procedure and uncertainty analysis are described in Section S1 of the Supporting Information S1.

The absorption‐dominated regime allows us to focus only on the material contribution to the quality factor. For the 4.4 μm sphere, the quality factor extracted from the experimental spectrum in Figure 5a is $Q_{\mathrm{tot}}∼10^{2}$. Since the calculated radiative component is $Q_{\mathrm{rad}}∼10^{4}$, its contribution to Equation (1) is indeed negligible, and one can assume that $Q_{\mathrm{tot}}≅Q_{\mathrm{mat}}$. Hence, the measured quality factor across the entire spectrum quantifies the absorption. Using the full CL spectrum acquired with a 30 keV beam in the SEM (Figure S1a in the Supporting Information S1), we extract $Q_{\text{mat}}$ from the larger spheres for both TM and TE resonances at wavelengths above 470 nm, where the peaks are well resolved. Below 470 nm, the signal‐to‐noise ratio decreases, and the resonances cannot be reliably separated for linewidth extraction. To access $Q_{\mathrm{mat}}$ at shorter wavelengths, we perform a CL spectrum measurement with a 200 keV beam in the Hitachi HF2000 TEM, using a tangential electron trajectory $\left(r_{e}\approx R_{\mathrm{sphere}}\right)$ to enhance phase‐matched excitation of WGMs. At 200 keV, the electron velocity approaches the phase velocity of the optical mode, enabling efficient coherent CL generation (see schematic illustration in Figure 5d). This phase‐matching channel is strongly suppressed at 30 keV (by at least seven orders of magnitude in our calculation, Figure 5c). Indeed, the CL spectrum from the TEM shows a propensity for TM‐polarization. For microspheres prepared under comparable conditions and exhibiting similar surface quality (see Figure S6 in the Supporting Information S1), we combine the values of $Q_{\mathrm{mat}}$ extracted from the SEM and TEM into a unified dataset, from which we can interpolate a wavelength‐dependent $Q_{\mathrm{mat}}\left(\lambda \right)$.

**FIGURE 5:**
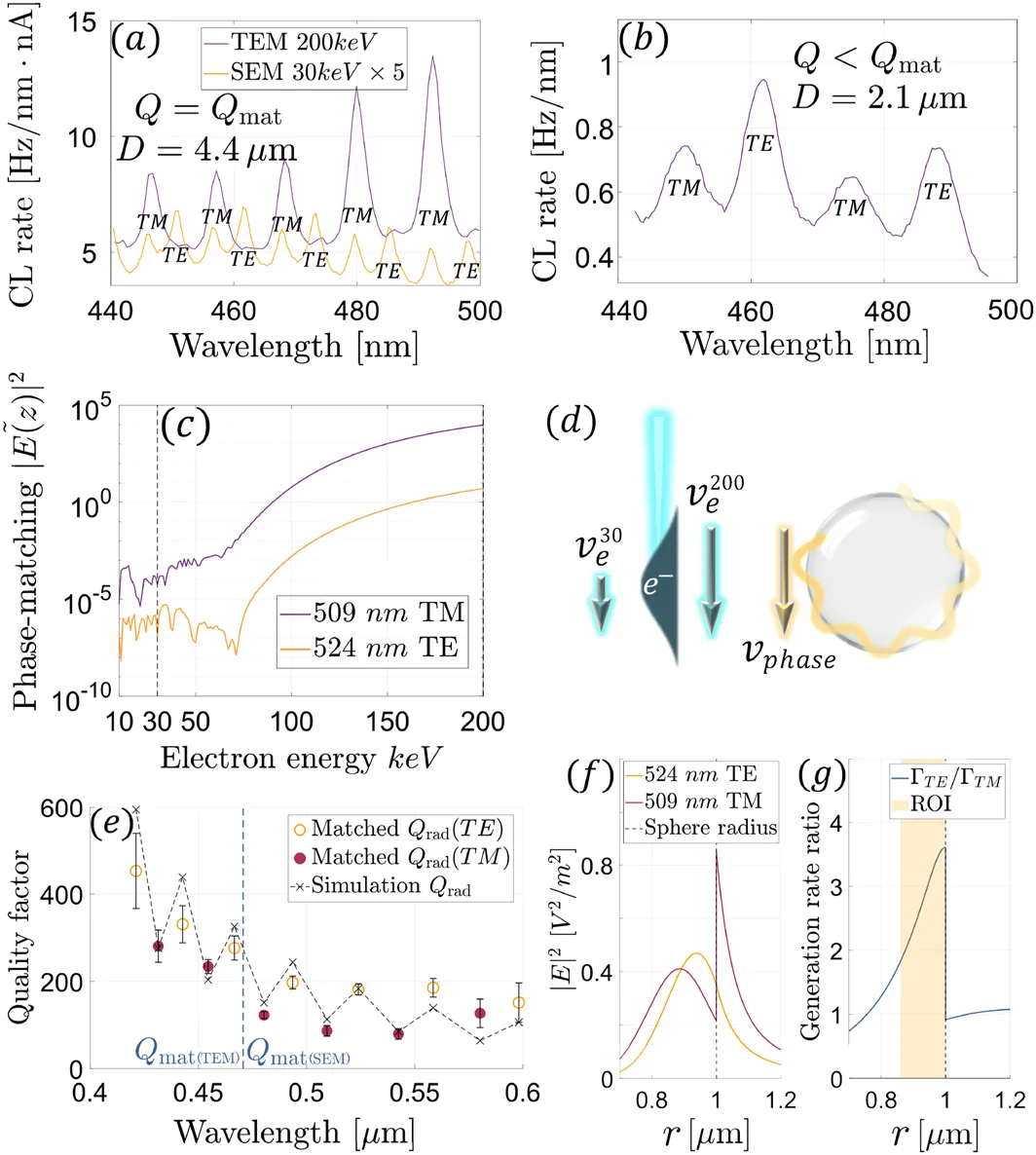
Phase matching trends and quality factor decomposition of silica microspheres. (a) CL spectra from a sphere with diameter D=4.4μm irradiated with electrons at 200 keV in a TEM (purple) and at 30 keV in an SEM (orange, scaled ×5). The TE/TM WGM resonances are marked, and $Q_{\mathrm{mat}}$ for each peak is extracted from a Lorentzian fit. (b) CL spectrum from a smaller sphere D=2.1μm (SEM, 30 keV). The resonances combine material loss and radiative leakage. (c) Calculated coherent CL efficiency for representative TM (509 nm) and TE (524 nm) modes. The dashed vertical lines indicate 30 and 200 keV. (d) Illustration of phase matching between the electron group velocities and the optical mode phase velocity. (e) Radiative quality factor $Q_{\mathrm{rad}}\left(\lambda \right)$ extracted using Equation (1). The experimental $Q_{\mathrm{rad}}$ for TE‐ and TM‐polarized modes (open‐ and filled‐circles, respectively) are compared to the simulated radiative losses (crosses, the dashed line is a guide to the eye). The blue dashed line marks spectral regions from which $Q_{\mathrm{mat}}$ was obtained using CL in the TEM (left) and in the SEM (right). (f) The calculated electric field modulus for TE‐ and TM‐polarized modes. (g) Local ratio of CL generation rates, $\Gamma_{TE}/\Gamma_{TM}$. The shading marks an estimated region of interest (ROI) of the interaction.

In the regime of balanced radiative and absorptive losses, as for the spheres with 2.1 μm diameter, the $Q_{\mathrm{mat}}\left(\lambda \right)$ measured in the larger spheres for both energies can be used within Equation (1) alongside the measured $Q_{\mathrm{tot}}$ to extract the purely radiative component, $Q_{\mathrm{rad}}$. The circle markers in Figure 5e show the extracted $Q_{\mathrm{rad}}$ for the resonances of the 2.1 μm sphere. The error bars are the standard deviation of $Q_{\mathrm{mat}}$ and $Q_{\mathrm{tot}}$. The quality factor decays for longer wavelengths as the angular modes are of a lower order. The TE‐polarized modes have a higher quality factor since they are more confined to the sphere, compared with the adjacent TM‐polarized modes. The black x‐markers are the radiative quality factors for the modes of the 2.1 μm sphere with a wavelength‐dependent refractive index around 1.45, simulated using the WGMode package [72]. The radiative quality factors extracted from the measurements agree well with the semi‐analytical toolbox predictions within the experimental uncertainties. The larger discrepancies of $Q_{\mathrm{rad}}$ at shorter wavelengths for the TE modes coincide with the increased systematic error in $Q_{\mathrm{mat}}^{TE}$. CL below 470 nm from the 4.4 μm spheres relies on coherent generation in the TEM so TE data were interpolated from the TM‐polarized peaks.

Although typically the CL‐photon generation depends on the electron energy, we use the calculated modes to assess that the photons in our experiment form strictly at the surface of the sphere. For the low‐energy electrons impinging on silica spheres, the emission is incoherent due to the lack of phase matching, and the electron velocity at 30 keV is $v_{e}\approx 0.3c$, whereas the optical phase velocity in silica is $v_{ph}\approx 0.69c$, where c is the speed of light. Moreover, we observe no clear signatures of transition radiation (TR) at 30keV, which would primarily contribute a broadband background rather than discrete peaks. Any TR is expected to be weak at this low electron velocity and is likely obscured by the stronger WGM‐related emission [26, 27]. Thus, the cathodoluminescence is mediated by material excitations, whose radiation according to Fermi's golden rule is governed by the local density of optical states (LDOS) [22, 73], which is the energy density of the WGM per photon [72]. The local photon generation rate Γ(ω,r) is proportional to

(2) $$\Gamma \left(\omega ,r\right)\propto \frac{|E\left(\omega ,\vec{r}\right){|}^{2}}{|E_{\max}{|}^{2}}\cdot \frac{\hbar \omega }{n_{\lambda }^{2}\epsilon_{0}V_{\text{eff}}}.$$

$E\left(\omega ,\vec{r}\right)$ is the modal electric field at position $\vec{r}$ and angular frequency ω, $\left|E_{\max}\right|$ is the maximum value of the Euclidean norm of the electric field, $n_{\lambda }$ is the refractive index of the mode at wavelength λ, $\epsilon_{0}$ is the vacuum permittivity, and $V_{\text{eff}}$ is the effective mode volume. We compare the total generation rate, Γ, for spectrally adjacent TM‐polarized and TE‐polarized modes, with central wavelengths of 509 nm and 524 nm, respectively. The ratio between the two polarizations traces‐out effects, such as out‐coupling, detector efficiency, and spectral response. Thus, it keeps the analysis purely in the photon generation physics and enables us to compare experimental parameters with their prediction from the semi‐analytic calculations. The measured CL counts from a particular mode, $N_{CL}$, depend on the generation rate and the probability of radiating rather than undergo absorption, $N_{CL}\propto \Gamma \frac{Q_{\mathrm{mat}}}{Q_{\mathrm{rad}}+Q_{\mathrm{mat}}}$. One can see that this form represents the competition between loss and radiation. In the limit of a non‐absorptive material, $Q_{\mathrm{mat}}\to \infty$, $N_{CL}\to \Gamma$, and all the generated photons radiate. In the opposite limit, $Q_{\mathrm{mat}}\to 0$, $N_{CL}\to 0$. Assuming $Q_{\mathrm{mat}}$ is similar for both TE and TM modes, in the poor material quality limit, the relative emission between TE and TM depends on their radiative rates, that is, if a photon manages to exit the cavity before it is absorbed, $\frac{N_{CL}^{TE}}{N_{CL}^{TM}}=\frac{\Gamma_{TE}}{\Gamma_{TM}}{\left(\frac{Q_{\mathrm{rad}}^{TE}}{Q_{\mathrm{rad}}^{TM}}\right)}^{-1}$. More generally, when $Q_{\mathrm{mat}}$ differs between TE and TM, we can extract the photon generation ratio, $\Gamma_{TE}/\Gamma_{TM}$, from our experimental observables, namely $Q_{\mathrm{rad}}$, $Q_{\mathrm{mat}}$, and the accumulated CL signal, $N_{CL}$ within the relevant spectral peaks, as follows:

(3) $$\frac{\Gamma_{TE}}{\Gamma_{TM}}=\frac{N_{CL}^{TE}}{N_{CL}^{TM}}\cdot \left(\frac{Q_{\mathrm{rad}}^{TE}+Q_{\mathrm{mat}}^{TE}}{Q_{\mathrm{rad}}^{TM}+Q_{\mathrm{mat}}^{TM}}\right)\cdot \frac{Q_{\mathrm{mat}}^{TM}}{Q_{\mathrm{mat}}^{TE}}$$

For the spectral peaks centered at 509 nm and 524 nm with an impact parameter of $r_{e}\approx R_{\mathrm{sphere}}$ (see Figure 2e), we find that $\Gamma_{TE}/\Gamma_{TM}=0.84\pm 0.25$, where the error represents the standard deviation of $\Gamma_{TE}/\Gamma_{TM}$ emanating from its multiple experimentally measured input variables. In the semi‐analytical calculation of the WGMode package, the ratio of $\Gamma_{TE}/\Gamma_{TM}$ exhibits a sharp discontinuity at the sphere's boundary, dropping from 3.5 to 1 inside and outside of the sphere, respectively (see Figure 5g). This behavior follows from the modal electric field profiles used in the calculation (Figure 5f), which show that the TE‐polarized mode dominates near the boundary since it is better confined [44, 72]. To enable a direct comparison with the experiment, we define a region of interest (ROI) corresponding to the electron excitation impact parameter, taken at a depth of 140nm from the surface of the sphere (see the shaded region in Figure 5g). It is a conservative estimate for an impact parameter of $r_{e}\approx R_{\mathrm{sphere}}-70\;nm$, and is symmetrically biased toward and away from the surface. In practice, the surface would contribute more. Averaging over this ROI, we find that ${\langle \Gamma_{TE}/\Gamma_{TM}\rangle }_{\mathrm{ROI}}\approx 2.7$. This value is higher by 7 standard deviations than our measurement of 0.84.

However, it seems that our measured photon generation ratios at 30 keV include an inherent contradiction. On one hand, the electron energy is too low for coherent CL (Figure 5c), allowing only CL mediated by excitations *in the material*. On the other hand, the measured ratio of the generation rates, $\frac{\Gamma_{TE}}{\Gamma_{TM}}=0.84\pm 0.25$, befits CL emission right *outside the sphere*, that is, in vacuum, where the calculated value is $\frac{\Gamma_{TE}}{\Gamma_{TM}}=0.93$. In other words, the CL is emitted from the material positioned in the vacuum. In reality, the sphere is imperfect and may exhibit some ovality or surface roughness (Figure S6 in the Supporting Information S1). Thus, the smearing of the interface between material and vacuum smooths the sharpness of the calculated transition of $\frac{\Gamma_{TE}}{\Gamma_{TM}}$. However, this seeming contradiction verifies that the *CL generation is located right at the sphere's boundary*. This surface sensitivity is granted here due to the edge‐localization of the optical WGMs, their edge field‐discontinuities, and the spectral separability of the TE‐ versus TM‐polarized modes. Typically, CL is dominated by the bulk, where the optical density of states at the surface is small, making the surface contribution negligible [22, 39, 74]. The surface sensitivity is further enhanced by the geometry of the near‐edge electron incidence. When the electron trajectory is tangent to the sphere $\left(r_{e}≲R_{\mathrm{sphere}}\right)$, it is parallel to the surface and remains superficial over hundreds of nanometers. Thus, the surface propensity of the electron incidence and the photonic modes enable the selective sensing of surface phenomena via CL without resorting to low electron energies [75]. This highlights the role of geometry and fabrication precision in shaping the CL response and sets the stage for engineering mode selectivity, polarization control, and directional output through careful tuning of the beam parameters and resonator dimensions.

## 4 Conclusions

In conclusion, we investigated cathodoluminescence in silica microspheres acting simultaneously as resonant cavities for optical whispering‐gallery modes and as collimating lenses for external emission. Although the various contributions to the CL are inherently convolved, we disentangle them by analyzing their spectral, spatial, and angular measurements. The properties of on‐sample lensing of the sphere for CL originating in its vicinity are characterized according to its angular directionality. For the nondirectional CL from WGMs, we apply a spectral analysis to disentangle photon generation, radiative out‐coupling, and absorptive loss mechanisms. Specifically, we find the quality factor related to material absorption and radiative whispering‐gallery loss, $Q_{\mathrm{mat}}$ and $Q_{\mathrm{rad}}$, respectively. This decomposition is used to quantify photon generation rates and deduce the surface sensitivity of WGM CL. Altogether, our results establish silica microspheres as model systems to probe electron‐photon interactions with mode selectivity while also serving as practical elements for improving the CL signal in advanced spectroscopy, exemplified by two particularly helpful aspects of nanoscopic photonics. First, it offers a path to investigate CL of surface electronic excitations by combining the edge‐parallel electron irradiation and surface photonic modes. Second, the on‐sample and high‐NA collimation by the sphere shapes CL into a low‐divergence and nearly diffraction‐limited beam and does so with high efficiency. Thus, it could enable low‐NA and otherwise constrained CL‐collection optics to produce a good spatial mode compatible with fiber coupling or quantum measurements [36, 61, 71].

## Author Contributions

All authors have accepted responsibility for the entire content of this manuscript and consented to its submission to the journal, reviewed all the results and approved the final version of the manuscript. H.A. prepared the samples, conducted the experiments alongside Z.B. in the SEM and S.M. in the TEM, analyzed the data, and prepared the figures. O.K. and H.A. wrote the manuscript with significant contributions from Z.B. and S.M. O.K. conceptualized and coordinated the research.

## Funding

This research was supported by The Israel Science Foundation (Grants 2992/24 and 1021/22). Ofer Kfir gratefully acknowledges funding from the Gordon Foundation, and from the Young Faculty Award from the National Quantum Science and Technology program of the Israeli Planning and Budgeting Committee. Hadar Aharon gratefully acknowledges funding from the National Quantum Science and Technology Program, the Center for Quantum Science and Technology at Tel‐Aviv University and the French NanoX project (ANR‐17‐EURE‐0009).

## Conflicts of Interest

The authors declare no conflicts of interest.

## Supporting information


Supporting Information S1

## Data Availability

The data that support the findings of this study are available from the corresponding author upon reasonable request.
