# Supplementary material for: Cathodoluminescence Enhancement Mechanisms in Silica Microspheres
Source: Nanophotonics. 2026 Mar 24;15(6):e70055. doi: 10.1002/nap2.70055 (PMC13642618; doi:10.1002/nap2.70055)
Supplement: Supplementary file 1 [file NAP2-15-e70055-s001.pdf]

Supplementary Material

Figure S1(a) compares cathodoluminescence (CL) spectra from a 4.4 μm diameter silica microsphere measured under 200 keV electron-beam excitation in a HF2000 TEM (Hitachi, purple) and 30 keV excitation in an Apreo 2S-LV SEM (Thermo Fisher Scientific, orange). Figure S1(b) shows a zoomed-in region of the 200 keV spectrum highlighting two resonances with Lorentzian fits.

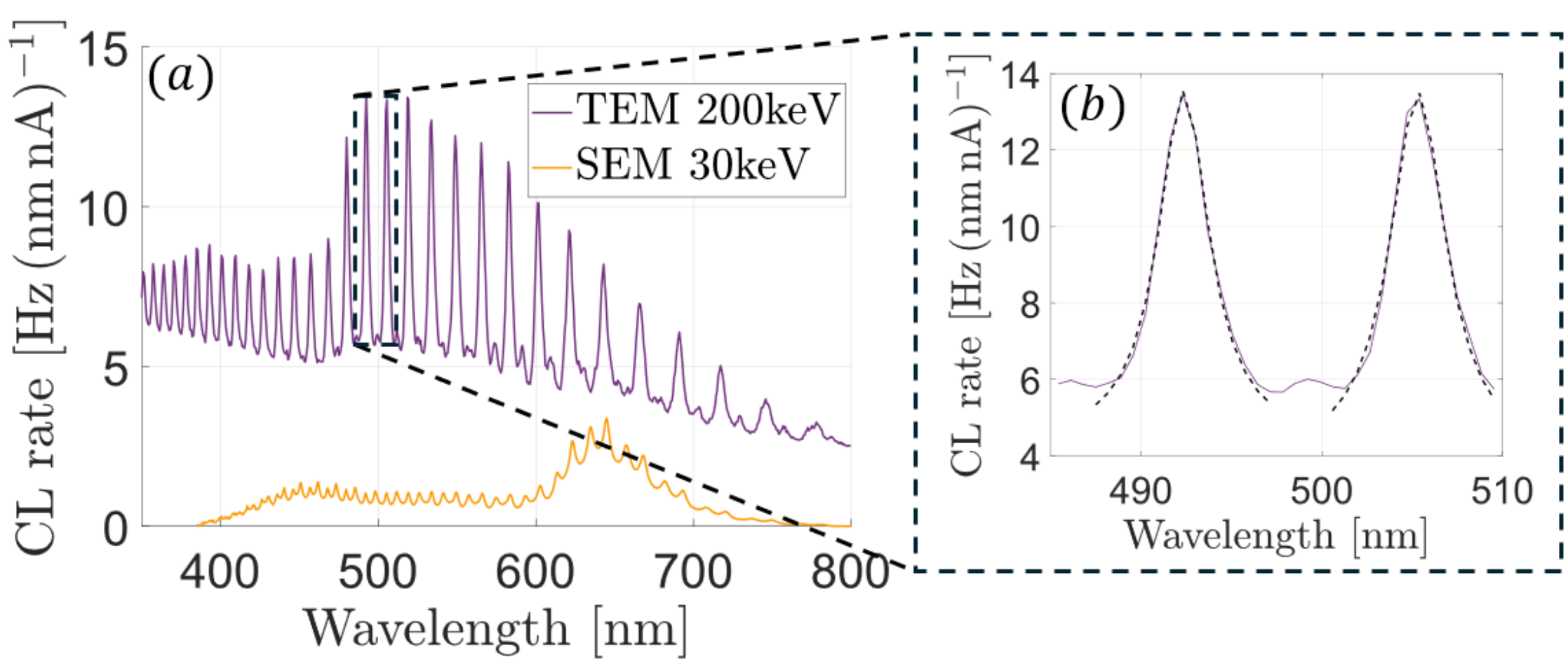

Fig. S1: (a) CL spectral rate from a 4.4μm diameter silica microsphere measured at 200 keV in a HF2000 TEM (purple) and at 30 keV in an Apreo 2S-LV SEM (orange). (b) Zoom-in of the 200 keV spectrum showing two representative resonances with Lorentzian fits (dashed).

1 Analytical extraction of Q_{mat} from CL spectrum

Each resonance in the CL spectrum is modeled by a Lorentzian line shape with a constant background,

$$I(\lambda) = \frac{A}{1 + \left(\frac{\lambda - \lambda_0}{\gamma}\right)^2} + C, \tag{1}$$

where A is the peak amplitude, λ_0 is the resonance wavelength, γ is the half-width at half-maximum (HWHM), and C is a constant offset. The full width at half maximum (FWHM) is

$$\Delta\lambda = 2\gamma. \tag{2}$$

The experimental quality factor of each measured mode is then obtained from

$$Q_{\mathrm{mat}} = \frac{\lambda_0}{\Delta\lambda} = \frac{\lambda_0}{2\gamma}. \tag{3}$$

Uncertainty of λ_0, $\Delta\lambda$, and Q_{mat}.
The Lorentzian parameters are determined by nonlinear least squares. Denoting the residual vector by $\mathbf{r}$ and the Jacobian of the model with respect to the fit parameters by $\mathbf{J}$, the residual variance is estimated as

$$s^2 = \frac{\sum_i r_i^2}{N - p}, \tag{4}$$

where N is the number of spectral samples used in the fit and p is the number of fitted parameters (here $p = 4$ for A, λ_0, γ, C). The parameter covariance matrix is approximated by

$$\mathrm{Cov}(\mathbf{b}) \approx s^2 \left(\mathbf{J}^{\mathsf{T}}\mathbf{J}\right)^{-1}, \tag{5}$$

where $\mathbf{b} = (A, \lambda_0, \gamma, C)^{\mathsf{T}}$. The standard errors are given by $\sigma_{b_k} = \sqrt{\mathrm{Cov}(\mathbf{b})_{kk}}$, yielding σ_{λ_0} and σ_γ. Propagating these to the linewidth and quality factor gives

$$\sigma_{\Delta\lambda} = 2\sigma_\gamma, \tag{6}$$

$$\sigma_{Q_{\mathrm{mat}}} = Q_{\mathrm{mat}} \sqrt{\left(\frac{\sigma_{\lambda_0}}{\lambda_0}\right)^2 + \left(\frac{\sigma_{\Delta\lambda}}{\Delta\lambda}\right)^2}. \tag{7}$$

Figure S2 presents a matrix measurement over 21 × 20 positions, where at each location the angularly resolved CL was recorded with a 10-second integration time and a 50 nm spectral filter centered at 550 nm. Fig. S2(a)-(f) show six representative examples from the matrix measurement.

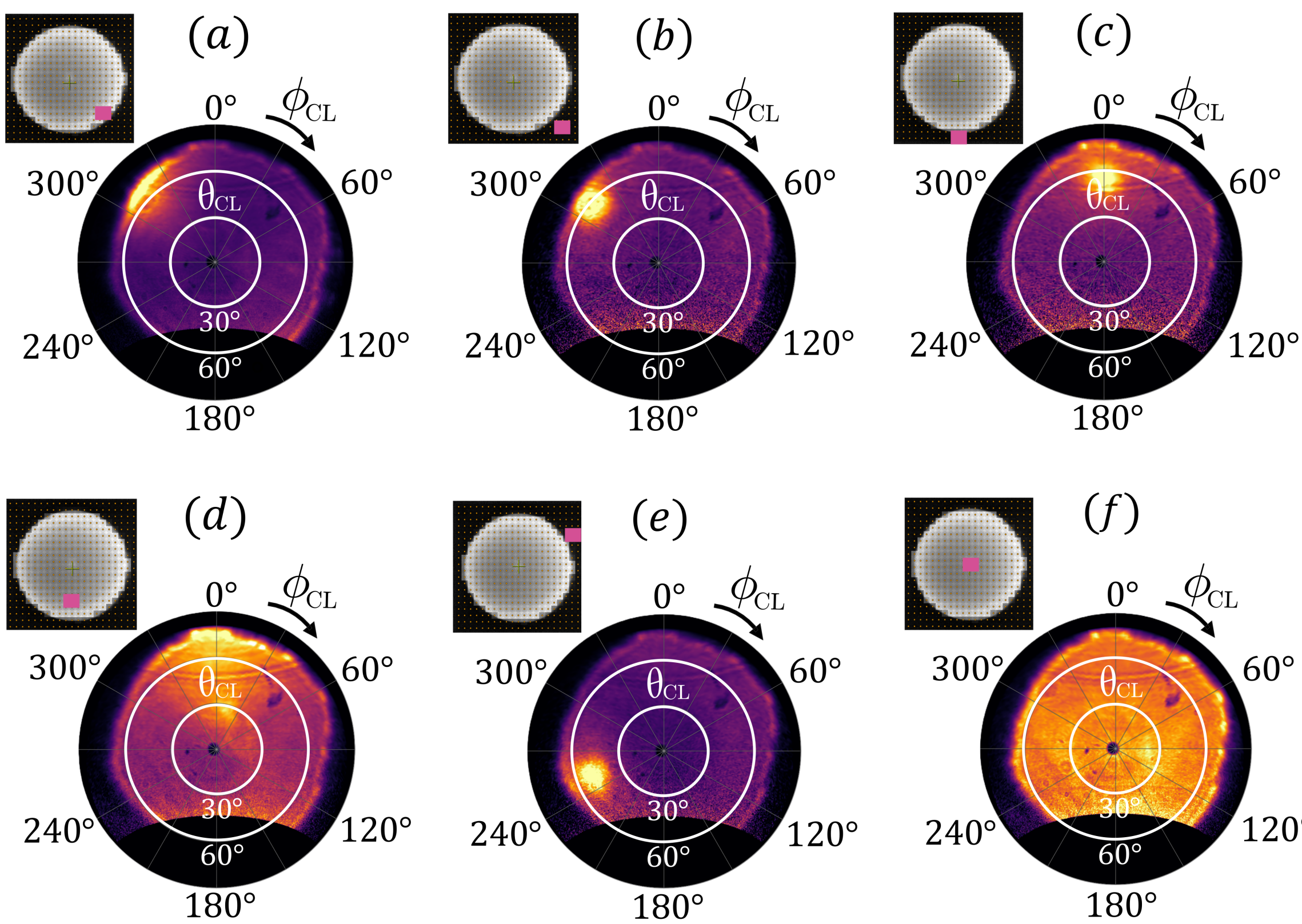

Fig. S2: (a)-(f) 2D maps showing the CL angular distribution from 2 μm diameter silica sphere at distinct electron excitation position, marked with pink square in each inset.

Figure S3 shows the CL spectra and corresponding angular emission patterns measured at five consecutive electron-beam positions along a line scan. The angular-resolved maps were acquired without a spectral filter, with an integration time of 5 seconds per map and per spectrum. Excitation positions outside the sphere show no detectable CL in the absence of an underlying layer, locations 4-5 exhibit negligible signal compared to location 3, where a carbon film is present.

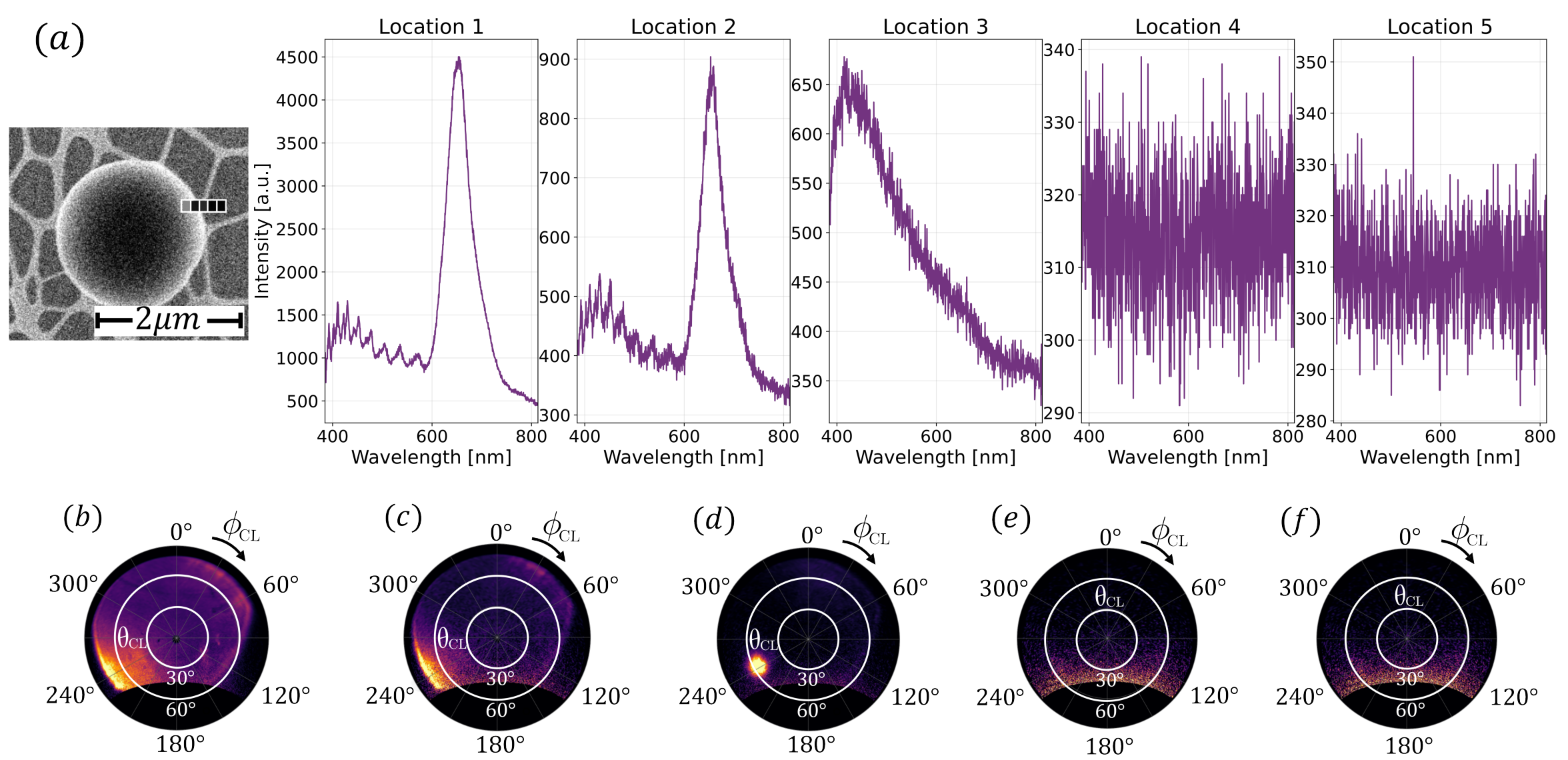

Fig. S3: CL spectra acquired from a 2 μm diameter silica microsphere at five electron-beam excitation positions. The positions are indicated by the five squares overlaid on the SEM image and correspond to locations (1)-(5) from left to right. (b)-(f) Corresponding 2D angular CL emission maps for locations 1-5, respectively.

Figure S4 presents θ-resolved CL spectrograms of 2 μm diameter silica-glass spheres, acquired with a 60-second integration time at different electron excitation positions. Note that these panels show the raw spectrograms and the θ_{CL} axis is plotted with the opposite sign convention compared to Fig. 2(d) in the main text, where negative angles appear at the bottom.

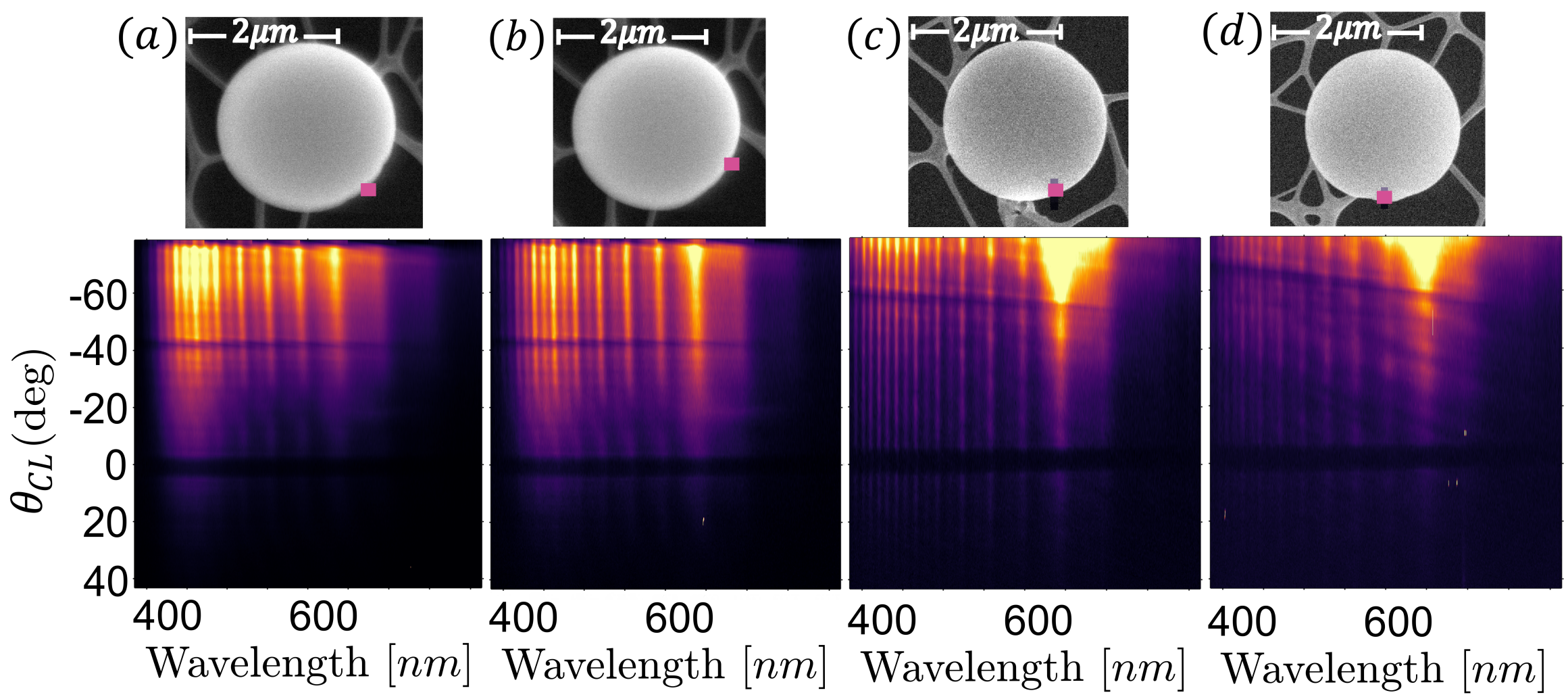

Fig. S4: θ-resolved CL spectrograms from 2 μm diameter silica spheres for different electron excitation positions, indicated by pink squares in the insets. (a–b) spectrograms from the same 2 μm sphere at two distinct excitation positions. (c–d) spectrograms from two different 2 μm spheres, each with a distinct excitation position. All plots exhibit WGM peaks with spatially varying backgrounds.

Figure S5 illustrates a geometrical-optics ray tracing model [1] for a point emitter located outside a spherical lens (radius R_0, refractive index $n = 1.45$). The simulation visualizes how refraction at the sphere interface focuses the emitted rays into a forward-directed angular distribution.

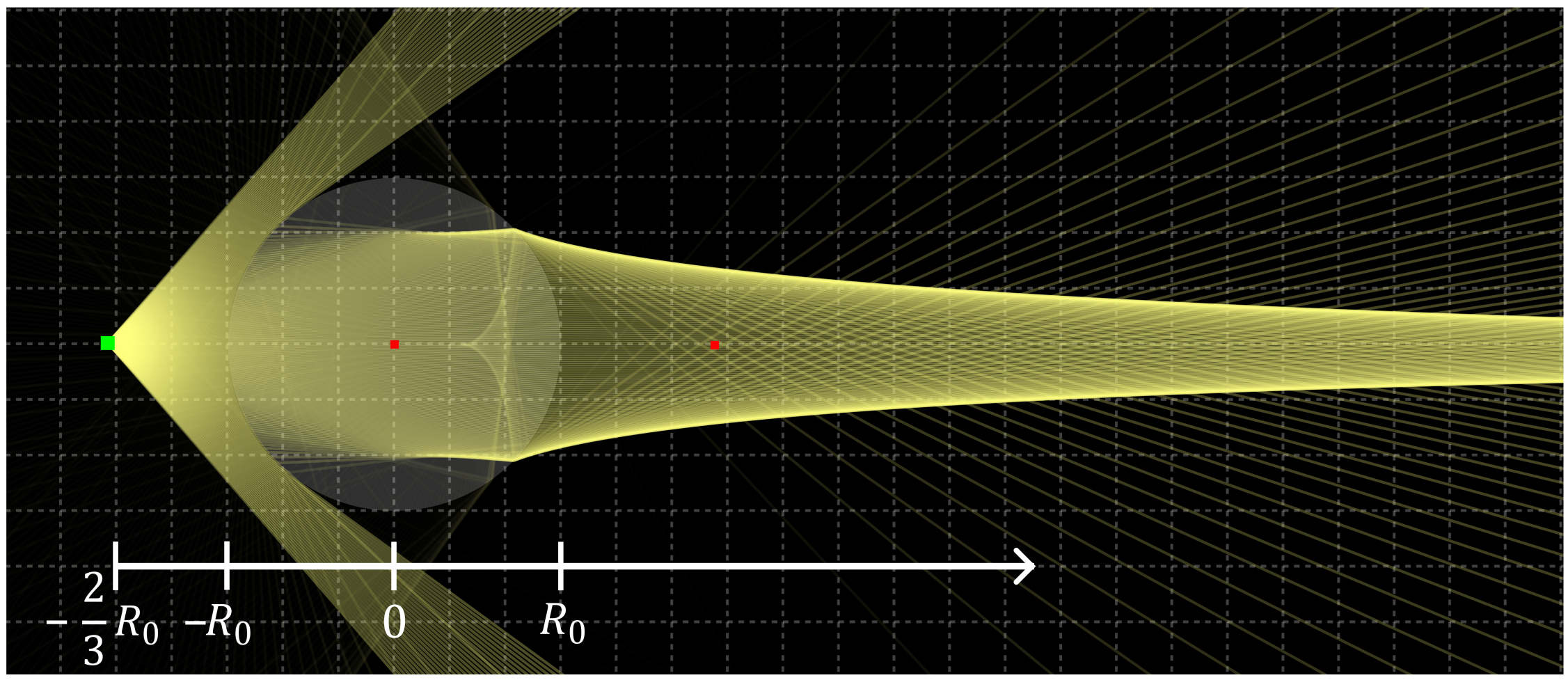

Fig. S5: Geometrical-optics ray tracing for a point source outside a spherical lens (radius R_0, refractive index $n = 1.45$), illustrating refraction and forward-directed ray redistribution. Generated using the open-source *Ray Optics Simulation* tool [1].

Figure S6 quantifies the surface roughness of 2 μm and 4.5 μm silica spheres from high-magnification SEM images of the rim. For each sphere, we extract the edge contour, fit an ideal circle of radius R_{fit}, and plot the radial deviation $\Delta r(\varphi) = r(\varphi) - R_{fit}$ versus azimuthal angle. The RMS roughness is defined as the standard deviation of $\Delta r(\varphi)$ and is reported in panels (c) and (e). We note that the extracted RMS values are comparable for the two sphere diameters.

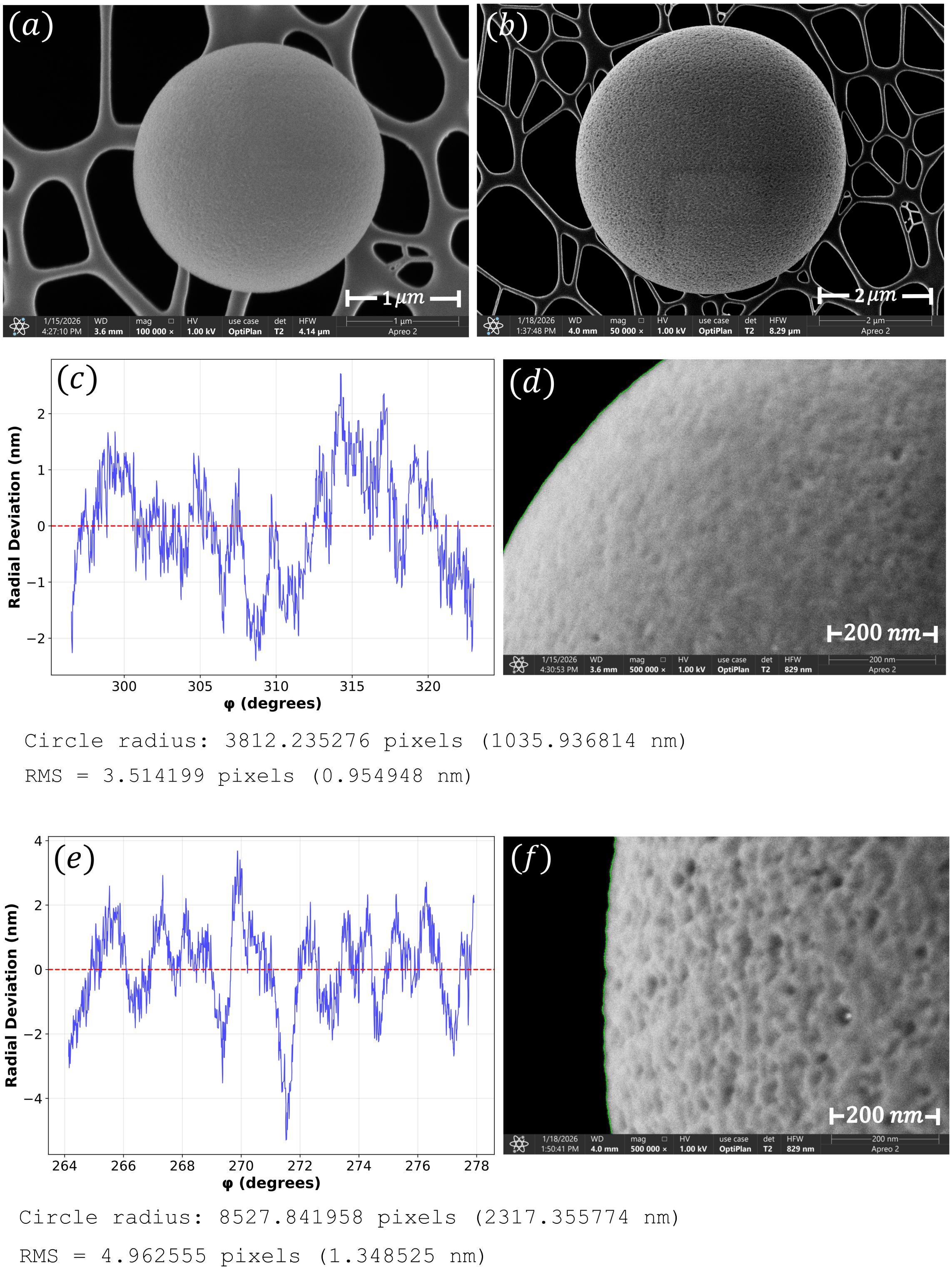

Fig. S6: Surface roughness characterization of silica microspheres. (a)-(b) Representative SEM images of spheres with 2 μm (a) and 4.5 μm (b) diameters. (c) The radial deviation from an ideal circle, $\Delta r(\varphi) = r(\varphi) - R_{fit}$, is plotted versus azimuthal angle φ (the red dashed line shows $\Delta r = 0$). (d) A high-magnification SEM image of the 2 μm sphere rim (200 nm scale bar) used to extract the local edge profile (green contour). The RMS roughness is taken as the standard deviation of $\Delta r(\varphi)$ and is indicated in the panel. (e)-(f) Same analysis for the 4.5 μm sphere: extracted edge profile (f) and corresponding radial deviations $\Delta r(\varphi)$ (e), yielding a similar RMS roughness as the $2\mu m$ sphere.

References

[1] Tu, Yi-Ting *et al.*, *Ray Optics Simulation* (2016), https://phydemo.app/ray-optics/.
